# ‘We're the Eyes and Ears… out There’: A Qualitative Exploration of the Perceptions and Experiences of Polypharmacy Amongst Home Care Providers

**DOI:** 10.1111/hex.70726

**Published:** 2026-06-14

**Authors:** Anum Iqbal, Liam Mullen, Adam Todd, Barbara Hanratty

**Affiliations:** ^1^ School of Pharmacy, Faculty of Medical Sciences Newcastle University Newcastle Upon Tyne UK; ^2^ Newcastle Patient Safety Research Collaboration (PSRC) Newcastle University Newcastle Upon Tyne UK; ^3^ Pharmacy Department Northumbria Healthcare NHS Foundation Trust Newcastle Upon Tyne UK; ^4^ Population Health Science Institute, Faculty of Medical Sciences Newcastle University Newcastle Upon Tyne UK

## Abstract

**Introduction:**

Polypharmacy is often managed at home by older people and those involved in their day‐to‐day care. Home care workers are well placed to play a crucial role, but there is little research in this setting. This study aimed to explore experiences and perceptions of polypharmacy amongst providers of home care.

**Methods:**

We conducted qualitative interviews with 15 home care staff from five for‐profit home care providers in three regions of England. Data were analysed thematically.

**Results:**

We identified three overarching themes: (1) managing medications within a fragmented care system, (2) decision‐making and autonomy and (3) client and family expectations and emotional burden of home care work. People providing home care services were striving to support older people with medications with limited training, communication or authority. The emotional toll on the home care workers was considerable.

**Conclusion:**

Home care supports an ageing population to live and be cared for, close to home. There are potential benefits to the management of polypharmacy, of better integrating home care into the broader health and care system. Clarity over roles, appropriate channels of communication and enhanced training are required.

**Patient or Public Contribution:**

Individuals with lived experience of home care services contributed meaningfully throughout the study. They advised on the development of the topic guide to ensure that interview questions were relevant, acceptable and reflective of real‐world home care experiences. At the mid‐point of data collection, public contributors reviewed insights and provided feedback on whether additional issues or perspectives should be explored in subsequent interviews. Following completion of data collection, they were also involved in discussing the preliminary findings and ensuring that the interpretation accurately reflected the experiences of those using and or accessing the service.

## 1 Introduction

Rising levels of polypharmacy, defined as the use of five or more medications, is a global concern [1, 2]. In many cases, polypharmacy is required to manage long‐term conditions, but the potential for harm (e.g., from drug–drug interactions, non‐adherence and adverse drug reactions) is high amongst older people [3, 4, 5]. Polypharmacy is also burdensome for older people and for those involved in their day‐to‐day care at home. Problematic polypharmacy refers to situations where the potential harms of multiple medicines outweigh the benefits for the individual, for example, due to adverse effects, interactions or treatment burden. Reducing problematic polypharmacy is therefore one of the most significant prescribing challenges, especially in primary care [6].

Polypharmacy has been studied in a wide range of healthcare settings [6, 7], but the focus has seldom been on the home, where the majority of older people age. Much support for older people to live independently is provided by unpaid family and friends, but an increasing number receive paid‐for home care [8]. In the United Kingdom, home care services have almost a million older adult clients [8], and this is predicted to increase by almost 60% in the next two decades [9]. Older people receiving home care are more likely to be living with frailty, multiple long‐term conditions and disability, which increase their vulnerability to medication‐related harm [10, 11]. In England, home care refers to paid support delivered in a person's own home to assist with personal care and activities of daily living. Services are commissioned by local authorities or purchased privately by individuals and their families. Whilst support is provided for personal care and activities of daily living, in most countries, home care workers are not health professionals [8, 10]. This means that they do not take on tasks judged to be within the remit of healthcare professionals, unless responsibility is delegated to the home care service. Nevertheless, administering medications, monitoring adherence and escalating concerns about potential adverse effects may all fall to the home care worker, as they are often there, in the home, when the needs arise.

A recent scoping review on polypharmacy and home care [12] identified a paucity of research in this topic area. Some suggestions for enhancing medication quality in home care were identified along with many gaps. Given the increasing reliance on home care and the limited knowledge of how medication is managed in this setting, this study aimed to explore the experiences, perceptions and views of polypharmacy among home care staff, using in‐depth semi‐structured interviews.

## 2 Methods

### 2.1 Design

A qualitative semi‐structured interview study, involving home care staff working in England, underpinned by reflexive thematic analysis (RTA) [13, 14].

### 2.2 Recruitment and Sampling

Participants were home care staff (managers or direct care providers), working in England. Home care providers were identified from public directories, the research team's existing contacts and by snowball sampling. We also used social media to recruit participants. A purposive maximum sampling approach was employed to ensure variation in age, experience (including client characteristics and service context) and geographical region served. Home care staff were also sampled according to their levels of experience and training. Each organisation was approached and invited to circulate the study details to their staff. Potential participants were invited to contact the research team who provided further written information on the research study. The researcher conducting the interviews (A.I., a female pharmacist with expertise in qualitative research methodology) was available to address any questions or concerns about the study. There were no existing relationships between the research team and any participants. Eligibility was confirmed during the consent process. Participants were asked to describe their role, employer and responsibilities. Where recruitment occurred via social media, work email addresses or appropriate work identification were requested to verify employment in home care. Individuals not currently working in home care in England or those unable to verify employment were excluded.

### 2.3 Data Collection and Analysis

In‐depth semi‐structured interviews were conducted by one researcher (A.I.) between April 2024 and October 2024. A topic guide was developed based on the existing literature [9, 15], piloted and refined with individuals with knowledge of and experience in the sector, focusing on experiences, understanding and views on multiple medication use and family/client expectations. Interviews were conducted via video conferencing facility (Zoom or Teams), telephone or in person, depending on the participant's preference. Interviews were conducted between the interviewer (A.I.) and the participant only. The interviews were audio recorded and transcribed verbatim. Notes were taken during interviews and incorporated into the analysis. Transcripts were reviewed iteratively by the research team during data collection to explore emerging concepts in subsequent interviews. Data collection continued until interviews were no longer generating new ideas or concepts. RTA, as detailed by Braun and Clarke [16], was used to identify recurrent themes, based on meaningful observations across the interviews using an inductive approach. The analytic process involved familiarisation with the data, generation of initial codes, development of themes, reviewing and refining themes, defining and naming themes and producing the final narrative. Initial coding was carried out by A.I., with regular discussion with the research team (B.H., L.M. and A.T.) to review interpretations and refine themes. A data coding tree was developed to illustrate the coding structure (see Supplementary File S1). NVivo (version 14) was used for data management. The consolidated criteria for reporting qualitative research checklist (COREQ) [17] was followed for this work (see Supporting File S2).

### 2.4 Ethical Approval

This study was given research ethics approval by Newcastle University Ethics Committee (reference 44321‐2024). All participants provided written informed consent to take part in the study.

## 3 Result

### 3.1 Participant Characteristics

**Figure 1:**
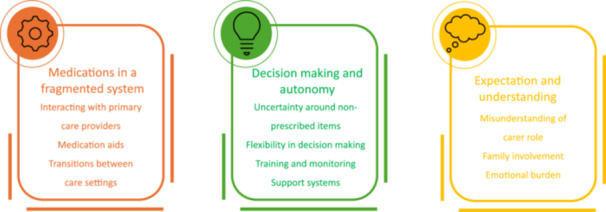
Overview of themes and subthemes emerging from interviews.

Fifteen home care workers were recruited and interviewed for the study (Table 1). Interviews ranged from 28 to 80 min in duration. The home care workers were recruited from five home care organisations, located across three regions of England (North East, South West and Midlands). All were for‐profit businesses. Three were national organisations (two of which were franchises) and three were smaller, regional providers (two in the north and one in the south east/west). Participants described the role they play in client care, including supporting clients to use their medications. From this, three themes with subthemes were identified (Figure 1) that reflected these experiences and views: (1) medication in a fragmented care system, (2) decision‐making and autonomy and (3) client and family expectation and the emotional burden of home care.

**Table 1:** Characteristics of interviewees.

Participant characteristics	Number	(%)
Gender		
Female	14	(93)
Male	1	(7)
Age in years		
< 30	2	(13)
30–39	3	(20)
40–49	5	(33)
50–59	2	(14)
60+	3	(20)
Region of England		
North East	6	(40)
West Midlands	4	(27)
South West	5	(33)
Years in Care Industry		
< 1	1	(7)
2–4	4	(27)
5–10	3	(20)
> 10	7	(46)

### 3.2 Theme 1: Medications in a Fragmented System

#### 3.2.1 Interacting With Primary Care Providers

Participants shared experiences of working across a fragmented care system, involving multiple organisations such as GP practices, community pharmacies and hospitals. In this context, it was perceived by the participants that there was limited communication or shared information between these organisations. This created barriers to communication and challenges of continuity of care, particularly when medication changes occurred or when clarification from clinicians was required. Indeed, many of them described medication‐related issues that were commonly encountered by participants, such as unclear prescription instructions, missing medicines or discrepancies in dosette boxes. When this occurred, home care staff were often physically separated from clinicians, pharmacists or other decision‐makers. Problems were therefore often first identified by care staff during visits in the home, and the interviewees provided examples of how they responded to, and ameliorated, medicated‐related problems.

I remember she couldn't because she still had like few tablets not to be crushed, but she couldn't swallow it. So, I had to ring the doctor. Again, and I said, look, she can't take this medication even its too small and whatever, so they give it to her in a liquid form.

(Participant 8)

In many cases, once the home care workers had identified a problem, they needed to interact with primary care providers such as community pharmacies or GP surgeries. Many interviewees reported difficulties in eliciting the timely support they needed from healthcare colleagues to rectify a problem.

So the amount of times we've had to speak to the pharmacy. Speak to the GP, regarding this medication.

(Participant 2)

… You know, the pharmacies and things are that busy. … just trying to get somebody to answer the phone, can be a nightmare… You know the joys of ringing GP surgeries.

(Participant 1)

Yes. I think they are helpful if they are contacted …., sometimes [surgeries are] too slow to respond…

(Participant 11)

#### 3.2.2 Medication Aids

All participants reported the use of medication aids, such as dosette boxes, supplied from a community pharmacy, to reduce the burden of administering multiple medication to clients:

From a carer's experience it's a lot easier at being in, like, a blister pack because everything's in one.

(Participant 7)

However, whilst aids were often helpful, interviewees were vigilant to errors with the set‐up.

So, her evening medication was in the morning and her morning was in the evening. So we've, we've had our fair share of problems with dosette boxes. One particular lady they [pharmacy team] keep putting on the wrong description on her medication.

(Participant 13)

Not that I'm picking on pharmacies, but a lot of times I have to ring a pharmacy. Because they've put the wrong medication in the wrong time slot or there's medication where it's alternative days and all of a sudden next week it's every day, and then sometimes fell out the tea call and put it in the morning call or just something like that. So I always double check everything I'm doing.

(Participant 5)

#### 3.2.3 Transitions Between Care Settings

Several participants described the problems that could occur when clients moved between care settings—almost all were concerned with communication. In some cases, home care providers were not informed about people returning home, leaving the client without a required, timely visit.

We had a gentleman that we were waiting for him to come home. Carer happened to pop in, and he had been there since…2:00 in the morning, but we never got there till lunchtime. Because we weren't aware that he was at home.

(Participant 13)

Medication changes in hospital were not always communicated to the home care provider in a timely manner or the changes reflected in the discharge medication.

… [The hospital] had sent a service user home and discharged them with the wrong medication.

(Participant 6)

They sent him home with completely different meds or just a bag of meds, and it's completely different to what we previously had. So that comes back to the office. And we spend a lot of time chasing the hospital.

(Participant 7)

Home care providers also provided examples of where their vigilant behaviour and knowledge of their clients gained over time, had positively impacted on patient safety for home care clients,

So she's [on] bisoprolol, omeprazole, edoxaban, apixaban and I go, No. No. … The apixaban is not to be given, but. I know the thing behind it and I knew that…. Had it come through to somebody else that isn't aware of that, then we could have very easily overdosed her on blood thinners.

(Participant 1)

### 3.3 Theme 2: Decision‐Making and Autonomy

#### 3.3.1 Uncertainty Around Non‐Prescribed Items

Working practices relating to prescribed medications were clear and understood by our participants. Decisions about administering items purchased by the client were an area of greater uncertainty. Whilst most believed that they were familiar with their clients' medications, caution was expressed about dealing with anything that was not prescribed. In many cases, the purchased items presented little risk to the client or care worker, and would not be considered as medicinal products (e.g., barrier creams).

For example, I had to get permission for using body lotion…So when she asks me, I can apply it.

(Participant 4)

Unless it is on a prescription, we will not administer that medication.

(Participant 6)

…if it's not on [the client's record], we're not allowed to administer any sort of medication.

(Participant 3)

Home carers were more certain in giving medication to clients when a health professional had authorised it and provided clear instructions on its use.

So as long as it's GP'd, it's dated. It's instructed and it's got the details of the client on that you're going to. Then you can give that medication.

(Participant 5)

#### 3.3.2 Flexibility in Decision‐Making

Some participants reflected that flexibility in responding to day‐to‐day care and treatment needs would be helpful, particularly in allowing for better client care.

…And if I got a customer that had a pressure sore on their bottom, for example, and they're soiled. You can't leave a dirty dressing on there and. You can ring the district nurses, but they could take a few hours to come out…

(Participant 13)

In some situations, home care workers felt that their role in the client's home and their detailed knowledge of the clients enabled them to identify potential care issues early. They believed these observations should be acknowledged when decisions are made about the client's care.

…because [we are] the eyes and ears… out there…

(Participant 4)

#### 3.3.3 Training and Monitoring

Training went some way to promote feelings of confidence and autonomy amongst care staff. Participants felt that they were well supported and appreciated structured training, with regular updates. However, this did not always cover medications, and a number of interviewees highlighted a need for more hands‐on learning in this area.

We do 3 days of full training, which is quite intense because we do the full days and we'll do what you call e‐learning and and it's always updating e‐learning.

(Participant 5)

If there's more money to put behind the training side, we would definitely focus more on medication, but it's just the resources. (Participant 7)

I feel confident we get quite a lot of it And the different ways …we get spot checked… we've got staff meetings talking about changes and updates it… So I would say I had enough training.

(Participant 10)

Regular spot checks were used in some organisations to monitor quality of care. The spot checks involved managers making unannounced visits to observe staff in clients' homes. Whilst this may be perceived as intrusive or intimidating, most home care workers in this study accepted it as part of maintaining high standards of care. One participant noted the benefits of these checks and knowledge sharing practices.

…and the different ways you know… we get spot checked, we share good practice.

(Participant 10)

#### 3.3.4 Support Systems

Formal support for home care workers is often provided by staff located in a centralised office, giving a central point of contact and easy access to assistance. Participants emphasised the value of knowing they had someone to turn to for help—this was particularly important in relation to questions about medications. New staff underwent a period of shadowing, where they were paired with senior colleagues to gain practical experience. This was widely regarded as a positive initiative. In addition to formal support structures, peer‐to‐peer assistance also played an important role. For example, WhatsApp groups were used among care staff to quickly communicate problems or concerns, creating an informal support network.

The office numbers we have two offices I have me managers mobile and I have all the care coordinators mobiles, so I'm never in that circumstance and if you couldn't and you were in the situation where you can't get a hold of anyone.

(Participant 5)

### 3.4 Theme 3: Client and Family Expectations and the Emotional Burden of Home Care

#### 3.4.1 Misunderstanding of Carer Role

Participants frequently encountered challenges relating to the expectations of client and family members about medication management in the home. Families were perceived to have limited awareness of where professional responsibilities begin and end. Their expectations that home care workers would take on responsibilities beyond their defined roles were sometimes accompanied by a belief that home care workers have greater medical authority than they actually do.

Families and clients are occasionally expecting us to basically fill the gaps without telling us what those gaps are…. Sometimes people are not really aware of what is …our responsibilities. They kind of expect us to to pick up everything else that nobody else picked up.

(Participant 10)

Yeah, you do get a lot where the families I don't know. The families think you're the doctor.

(Participant 5)

#### 3.4.2 Family Involvement

Demands from local authority social services could also seem unreasonable if they were not fully accounting for time constraints. In this context, supportive family members who took an active role in medication management were appreciated.

And then social services tell us what is from their point of view, important to do in half an hour visit.

(Participant 8)

We just contact the son, and he's golden. He goes and collects the dosette boxes and goes straight back to the pharmacy with them. Yeah, he's really good.

(Participant 13)

I have one service user whose family is very, very, very attentive when it comes to medication.

(Participant 14)

#### 3.4.3 Emotional Burden

Participants highlighted the emotional toll of high workloads, while also emphasising the dedication and professionalism of home carers. Workload was a significant challenge reported by home care providers across all interviews. While participants acknowledged being busy throughout their working day, they were keen to emphasise that the quality of care should not be compromised despite time pressures. Many carers described feeling overwhelmed by the number of tasks they were required to complete. This could lead to emotional distress, particularly when they were unable to fully meet client and family expectations. Participants highlighted how the dedication of carers sometimes led to delays in their schedule:

…and that puts pressure on the care staff because some of them won't leave that call until they've done everything that they've been asked to do, which then has a knock‐on effect to the next call because they're late and so forth.

(Participant 13)

Another participant reflected on the weight of responsibility involved, particularly in the management of client medication:

…there's a lot of priorities. There's a lot of pressure. There's a lot of responsibilities dealing with medication.

(Participant 2)

You're also meant to know how 1 drug would react with another drug that they're on. And I personally feel that that's impossible for people to know unless you're in the industry, whereas we're I don't want to say we're just carers because we're not just carers. Yeah, it's a lot of responsibility.

(Participant 15)

Despite these pressures, participants emphasised the compassion and commitment that underpinned their role, and the value they placed on supporting clients over time.

…whoever has chosen this role, they must have chosen it because they do care for the people. Actually, apart from the the job, they do actually care for the people by heart and it is, it is hard to see people struggling with something … and especially when you going to them regularly …you you really feel like feel for the people.

(Participant 9)

## 4 Discussion

This study explored views, perceptions and experiences of polypharmacy amongst home care providers. People providing home care services were passionate about caring for their clients, and the interviews provided insights into the consequences of working under pressure in a fragmented system. Staff autonomy was perceived to be constrained, and there was often conflict between meeting client and family expectations, particularly making decisions about medications or administering products that were not prescribed. The emotional toll on the home care workers was not insignificant.

### 4.1 Comparison With Other Work

Previous studies have described the increased risk of medication errors and adverse drug events among home‐dwelling older adults associated with polypharmacy [4, 5, 18]. This study, and our recent scoping review [12], suggest that home care staff may be an important but overlooked part of the response to this problem, providing a safety net, detecting errors and communicating with prescribers about potential problems. However, we found that the ability of home care workers to intervene effectively may be hindered by restricted access to prescribers, echoing a wider literature that suggests polypharmacy‐related challenges are often exacerbated system‐wide fragmentation and poor interprofessional communication [12, 19, 20, 21]. Our home care participants perceived ambiguities between their roles and those of healthcare professionals, particularly in relation to administering medications, access to emergency supplies and responding to side effects. Previous research has also identified a widespread misconception that home carers have clinical training, resulting in discrepancies between service users' expectations and the actual scope of care provision [22].

### 4.2 Strengths and Limitations

Our findings provide novel insights into the role of care workers in medication management, the process of ensuring that medicines are used safely, effectively and appropriately to achieve the best possible health outcomes. This is a challenging area for research, where the nature of care work and time pressures faced by staff present potential barriers to research participation. Despite this, we recruited interviewees from a range of geographical locations, expertise and organisational structures. We acknowledge that our findings may not be directly relevant to places with different home care systems and regulatory frameworks. We also focused solely on carer perspectives and gained no direct insights into patient or family experiences. Patient and Public Involvement and Engagement (PPIE) partners helped to shape interview topics, but the challenges faced by clients and their families in polypharmacy management remain to be explored in future research.

### 4.3 Implications

Clarity over the role of home care in healthcare‐related tasks is vital, given the broader international shift in home care, to delegate tasks from health to social care [23]. In the United Kingdom, a move to neighbourhood‐based models of care [24], with services provided closer to home, emphasises the importance of clearly defined roles and responsibilities in this context. Having clearly defined roles and responsibilities will benefit both patient/client safety and staff wellbeing. Indeed, role ambiguity has been previously reported in other professional groups, such as social workers [25], and is linked to poor job satisfaction and retention [26]. Specific attention is also required to affording home care workers some discretion to safely administer treatments that clients have purchased from pharmacies. This would support patient autonomy and self‐management and allow home care clients to retain independence.

This study suggests that practical opportunities to learn about medicine administration would be welcomed in home care. Current training programmes often focus on e‐learning due to workforce constraints, with limited exposure to real‐world medication challenges. Evidence from healthcare settings has demonstrated that simulation‐based training effectively enhances staff confidence and competence in medication management by replicating real‐life scenarios in a controlled environment [27]. Medication errors are known to be common in‐home care settings [28, 29]; strengthening access to a broader range of training opportunities for home care staff may have a key role to play in promoting patient safety.

Communication across health and social care settings is a frequently cited source of poor care. In this study, participants described difficulties in communicating with GP practices, community pharmacies and hospitals, particularly when trying to resolve medication‐related problems or clarify discharge information. These experiences highlight the importance of accessible communication routes between home care providers and other parts of the health and care system.

Home care providers also described managing practical medication challenges on a day‐to‐day basis, including issues with dosette boxes, unclear prescription instructions and delays in receiving responses from healthcare professionals. These findings suggest that additional support for home care workers in managing aspects of polypharmacy may help alleviate pressure elsewhere in the care system.

## 5 Conclusions

Polypharmacy is a growing public health concern, particularly among older adults, who increasingly rely on home care services. Home care providers play a crucial role in medication management and will continue to be an integral part of care delivery. This study highlights the complexities faced by carers, including role ambiguity, the challenges of managing multiple medications and navigating expectations from both families and healthcare providers. The findings underscore the potential benefits of better integration of home care into the broader health and care system.

## Author Contributions

**Anum Iqbal:** conceptualization, investigation, methodology, validation, formal analysis, project administration, data curation, visualization, writing – review and editing, writing – original draft, resources. **Liam Mullen:** methodology, formal analysis, writing – review and editing. **Adam Todd:** writing – review and editing, methodology, supervision, funding acquisition, formal analysis, conceptualization. **Barbara Hanratty:** supervision, writing – review and editing, conceptualization, funding acquisition, formal analysis, methodology.

## Ethics Statement

This study was given research ethics approval by Newcastle University Ethics Committee (reference 44321‐2024). All participants provided written informed consent to take part in the study.

## Conflicts of Interest

The authors declare no conflicts of interest.

## Supporting information

Supporting File 1

Supporting File 2

## Data Availability

The data that support the findings of this study are available from the corresponding author upon reasonable request.
