# Supplementary material for: ‘We're the Eyes and Ears… out There’: A Qualitative Exploration of the Perceptions and Experiences of Polypharmacy Amongst Home Care Providers
Source: Health Expect. 2026 Jun 14;29(3):e70726. doi: 10.1111/hex.70726 (PMC13264679; doi:10.1111/hex.70726)
Supplement: Supplementary file 1 — Supporting File 1 [file HEX-29-e70726-s002.docx]

| **Supplementary Table: Coding tree illustrating the development of themes and subthemes** | | |  |
| --- | --- | --- | --- |
| **Theme** | **Subtheme** | **Codes** |  |
| Theme 1: Medications in a fragmented care system | Subtheme 1.1: Interacting with primary care providers | Code 1: Identifying medication problems during visits  Code 2: Feeling isolated from clinicians who prescribe medication  Code 3: Managing medication issues in the home environment  Code 4: Resolving problems without immediate clinical input |  |
|  | Subtheme 1.2: Medication aids | Code 1: Use of medication aids to support administration  Code 2: Discrepancies between prescriptions and blister packs  Code 3: Contacting clinicians to resolve medication aid discrepancies |  |
|  | Subtheme 1.3: Transitions between care settings | Code 1: Medication discrepancies on hospital discharge  Code 2: Lack of planning and notification when patients return home  Code 3: Poor communication of medication related changes  Code 4: Chasing hospitals or GP practices for information/clarification | |
|  |  |  |  |
| Theme 2: Decision-making and autonomy | Subtheme 2.1: Uncertainty around non-prescribed items | Code 1: Caution about how to manage over-the-counter products  Code 2: Needing permission to apply certain products  Code 3: Reliance on clinicians to authorise administration/usage |  |
|  | Subtheme 2.2: Training and monitoring | Code 1: Limited medication-specific training  Code 2: Reliance on e-learning  Code 3: Desire for more practical training through experience |  |
|  | Subtheme 2.3: Support systems | Code 1: Office-based support systems  Code 2: Role of care managers for advice  Code 3: Peer support systems among carers  Code 4: Informal channels of communication and support |  |
| Theme 3: Expectation and understanding | Subtheme 2.1: Misunderstanding of carer role | Code 1: Families assuming carers have clinical authority  Code 2: Unclear expectations about responsibilities  Code 3: pressurised to fill support gaps in care provision |  |
|  | Subtheme 2.2: Family involvement | Code 1: Supportive family role acknowledged and valued  Code 2: Assisting with medication collection from pharmacy  Code 3: Family expectations influencing delivery of care |  |
|  | Subtheme 2.3: Emotional burden | Code 1: Workload pressure  Code 2: Sense of responsibility for medication safety  Code 3: Challenge of prioritising care in limited time  Code 4: Emotional impact of caring and not meeting expectations |  |
