# Supplementary material for: ‘We're the Eyes and Ears… out There’: A Qualitative Exploration of the Perceptions and Experiences of Polypharmacy Amongst Home Care Providers
Source: Health Expect. 2026 Jun 14;29(3):e70726. doi: 10.1111/hex.70726 (PMC13264679; doi:10.1111/hex.70726)
Supplement: Supplementary file 2 — Supporting File 2 [file HEX-29-e70726-s001.docx]

| **Number** | **Item** | **Guide questions / description** | **Reported on manuscript page** |
| --- | --- | --- | --- |
| **Domain 1: research team and reflexivity** | | | |
| **Personal characteristics** | | | |
| 1 | Interviewer | Which author(s) conducted the interviews? | 3 |
| 2 | Credentials | What were the researcher’s credentials? *E.g., PhD, MD* | 3 |
| 3 | Occupation | What was their occupation at the time of the study? | 3 |
| 4 | Gender | Was the researcher male or female? | 3 |
| 5 | Experience and training | What experience or training did the researcher have? | 3 |
| **Relationship with participants** | | | |
| 6 | Relationship established | Was a relationship established prior to study commencement? | 3 |
| 7 | Participant knowledge of interviewer | What did the participants know about the researcher?  *E.g., reason for doing the research* | 3 |
| 8 | Interviewer characteristics | What characteristics were reported about the interviewer?  *E.g., bias, assumptions, reasons and interests in the research topic* | 3 |
| **Domain 2: study design** | | | |
| **Theoretical framework** | | | |
| 9 | Methodological orientation and theory | What methodological orientation was stated to underpin the study?  *E.g., grounded theory, ethnography, discourse analysis* | 3 |
| **Participant selection** | | | |
| 10 | Sampling | How were participants selected? *E.g., purposive, convenience, consecutive* | 3 |
| 11 | Method of approach | How were participants approached? *E.g., face-to-face, telephone, email* | 3 |
| 12 | Sample size | How many participants were in the study? | 4, Table 1 |
| 13 | Non-participation | How many people refused to participate or dropped out (with reasons)? | N/A |
| **Setting** | | | |
| 14 | Setting of data collection | How was the data collected? *E.g., home, clinic, workplace* | 3, 4, Table 1 |
| 15 | Presence of non-participants | Was anyone else present besides the participant and researcher? | 3 |
| 16 | Description of sample | What are the important characteristics of the sample? *E.g., demographic data* | 3, Table 1 |
| **Data collection** | | | |
| 17 | Interview guide | Were questions and prompts provided by the authors? | 3 |
| 18 | Repeat interviews | Were repeat interviews carried out? If yes, how many? | N/A |
| 19 | Audio/visual recording | Did the researcher use audio or visual recording to collect the data? | 3 |
| 20 | Field notes | Were field notes made during/after the interview? | 3 |
| 21 | Duration | What was the duration of the interviews? | 4 |
| 22 | Data saturation | Was data saturation discussed? | 3 |
| 23 | Transcripts returned | Were transcripts returned to participants for comment/correction? | N/A |
| **Domain 3: analysis and findings** | | | |
| **Data analysis** | | | |
| 24 | Number of data coders | How many data coders coded the data? | 3 |
| 25 | Description of the coding tree | Did authors provide a description of the coding tree? | Supplementary File 1 |
| 26 | Derivation of themes | Were themes identified in advance or derived from the data? | 3 |
| 27 | Software | What software, if applicable, was used to manage the data? | 3 |
| 28 | Participant checking | Did participants provide feedback on the findings? | N/A |
| **Reporting** | | | |
| 29 | Quotations presented | Were participant quotations presented to illustrate the themes / findings? Was each quotation identified? E*.g., participant number* | 6-12 |
| 30 | Data and findings consistent | Was there consistency between the data presented and the findings? | 6-12 |
| 31 | Clarity of major themes | Were major themes clearly presented in the findings? | 6-12, Figure 1 |
| 32 | Clarity of minor themes | Is there a description of diverse cases or discussion of minor themes? | 6-12 |
